# State Changes During Resting-State (Magneto)encephalographic Studies: The Effect of Drowsiness on Spectral, Connectivity, and Network Analyses

**DOI:** 10.3389/fnins.2022.782474

**Published:** 2022-06-14

**Authors:** Eva M. M. Strijbis, Yannick S. S. Timar, Deborah N. Schoonhoven, Ilse M. Nauta, Shanna D. Kulik, Lodewijk R. J. de Ruiter, Menno M. Schoonheim, Arjan Hillebrand, Cornelis J. Stam

**Affiliations:** ^1^Department of Neurology, MS Center Amsterdam, Amsterdam Neuroscience, Amsterdam UMC, Vrije Universiteit Amsterdam, Amsterdam, Netherlands; ^2^Department of Clinical Neurophysiology, Amsterdam Neuroscience, Amsterdam UMC, Vrije Universiteit Amsterdam, Amsterdam, Netherlands; ^3^Department of Anatomy and Neurosciences, MS Center Amsterdam, Amsterdam Neuroscience, Amsterdam UMC, Vrije Universiteit Amsterdam, Amsterdam, Netherlands

**Keywords:** magnetoencephalography (MEG), EEG, graph connectivity analysis, spectral power analysis, drowsiness

## Abstract

**Background:**

A common problem in resting-state neuroimaging studies is that subjects become drowsy or fall asleep. Although this could drastically affect neurophysiological measurements, such as magnetoencephalography (MEG), its specific impact remains understudied. We aimed to systematically investigate how often drowsiness is present during resting-state MEG recordings, and how the state changes alter quantitative estimates of oscillatory activity, functional connectivity, and network topology.

**Methods:**

About 8-min MEG recordings of 19 healthy subjects, split into ~13-s epochs, were scored for the presence of eyes-open (EO), alert eyes-closed (A-EC), or drowsy eyes-closed (D-EC) states. After projection to source-space, results of spectral, functional connectivity, and network analyses in 6 canonical frequency bands were compared between these states on a global and regional levels. Functional connectivity was analyzed using the phase lag index (PLI) and corrected amplitude envelope correlation (AECc), and network topology was analyzed using the minimum spanning tree (MST).

**Results:**

Drowsiness was present in >55% of all epochs that did not fulfill the AASM criteria for sleep. There were clear differences in *s*pectral results between the states (A-EC vs. D-EC) and conditions (EO vs. A-EC). The influence of state and condition was far less pronounced for connectivity analyses, with only minimal differences between D-EC and EO in the AECc in the delta band. There were no effects of drowsiness on any of the MST measures.

**Conclusions:**

Drowsiness during eyes-closed resting-state MEG recordings is present in the majority of epochs, despite the instructions to stay awake. This has considerable influence on spectral properties, but much less so on functional connectivity and network topology. These findings are important for interpreting the results of EEG/MEG studies using spectral analyses in neurological disease, where recordings should be evaluated for the presence of drowsiness. For connectivity analyses or studies on network topology, this seems of far less importance.

## Introduction

Quantitative analyses of neurophysiological data in terms of spectral changes and alterations in (functional) connectivity and network topology have changed our thinking on how neuropathology can lead to cognitive disability (Stoffers et al., 2007; de Waal et al., 2012; Schoonhoven et al., 2019). Complicating factors for such analyses are the influence of physiological changes that may occur during changes in condition or vigilance state. Although it may seem easy to control different conditions, such as instructing subjects to stay awake or to not open their eyes during eyes-closed recordings, evidence suggests the contrary. Subjects dose off easily, also in noisy environments, such as MRI scanners (Poudel et al., 2014; Tagliazucchi and Laufs, 2014). In the quiet EEG/MEG environment, this may be even more problematic. In addition, spontaneous opening of the eyes during EEG/MEG recordings occurs frequently, despite the instructions.

The spectral changes that both eye-opening and drowsiness or sleep induce have been recognized since the first recording of the EEG by Hans Berger in the first half of the twentieth century (Berger, 1929; Schomer and Lopes da Silva, 2005). Opening of the eyes leads to suppression of the dominant background pattern (the “alpha rhythm”). This alters the results of spectral analyses significantly (Glass and Kwiatkowski, 1970). Fortunately, the distinction between eyes-open and eyes-closed conditions can be made relatively easily with a visual inspection of the time series. It shows suppression of the posterior-dominant rhythm in the parietooccipital areas, often in conjunction with the presence of eye-blink artifacts. Sleep [non-REM 1 (NREM1)] is another phenomenon that can be relatively easy to assess with a visual inspection of time series. It is characterized by the diffuse slowing of the background rhythm which increases both theta and delta power in >50% of a 30-s epoch. These specific criteria for the EEG definition of sleep are defined in the American Academy of Sleep Medicine Guidelines (AASM) criteria (Berry et al., 2017). A more challenging problem is the more dynamic state change of impaired vigilance, or drowsiness. The detection of drowsiness is difficult since encephalographic patterns differ, it is often short and subjects are unaware of any drowsiness (Maulsby et al., 1966; Santamaria and Chiappa, 1987; Deuker et al., 2009). It precedes longer periods of oscillatory slowing and alpha dropout, but there can be significant increases in lower frequency band oscillations (Lal and Craig, 2002; Li et al., 2020).

The oscillatory slowing in drowsiness can theoretically influence consistency, reproducibility, and test–retest reliability of spectral power and connectivity measurements. Measurement validity in EEG and MEG has been the topic of numerous reports in healthy subjects as well as different neurological or psychiatric diseases. The consistency and test–retest reliability of spectral power estimates with EEG/MEG are generally good, even with low amounts of data and the number of subjects (Salinsky et al., 1991; Napflin et al., 2007; Marquetand et al., 2019). In fact, there seems to be a genetic heritability in the background pattern that underscores the robustness of the spectral power measurements (Smit et al., 2005). However, brain functional connectivity estimates depend on a number of factors, including amount of data, technical considerations in terms of filtering, artifact rejections, and other preprocessing steps and type of connectivity calculation used (refer to an extensive overview) (Jin et al., 2011; Hardmeier et al., 2014; Colclough et al., 2016; Garces et al., 2016; Pernet et al., 2020). Interestingly, the influence of state changes on graph theoretical measures has received even less attention than the effects of drowsiness on spectral power. In an MEG reproducibility study, Deuker et al. (2009) found a substantially lower ICCs for different graph metrics between repeated resting-state and task recordings, suggesting greater variation in resting-state recordings. While they argued that low reliability in the resting state might be a consequence of the diverse nature of neural patterns found in resting state, drowsiness was not taken into account here. Marquetand et al. (2019) showed that vigilance only has a relatively small influence on overall test–retest reliability of functional connectivity measures. However, they only compared selected awake epochs instead of the effects of drowsiness specifically. They also studied test–retest reliability and not the influence of state changes on connectivity measures *per se*.

Therefore, this study aimed to systematically investigate to what extent these state changes are present during resting-state MEG recordings, and how these alter quantitative MEG estimates of oscillatory activity, functional connectivity, and network topology in healthy controls. We screened a large dataset of healthy controls for the presence of sufficient data in the alert resting-state, drowsy resting-state, and eyes-closed conditions. Our main hypothesis was that vigilance changes are frequently present, and that they significantly alter spectral power similar to the effects of opening of the eyes. Following from this, we hypothesized that connectivity estimates are also significantly influenced by state and condition. If this is indeed the case, careful selection of resting-state data is of greater importance for studies into MEG-related biomarkers, being either spectral power measurements or connectivity analysis than now generally assumed. Not only should data selection then focus on artifacts or clear sleep, but also on unexpected protocol deviations (eyes-opening) or drowsiness. Specifically, when MEG measures, such as oscillatory slowing or altered, functional connectivity and network properties, are studied as a potential biomarker for disease.

## Methods

### Case Selection

Existing resting-state MEG recordings of 66 healthy controls were selected from the larger Amsterdam multiple sclerosis (MS) cohort. The Amsterdam MS cohort is a prospective cohort that is focused on finding imaging and biomarker determinants of disease progression in MS. Visits consist of a clinical assessment of neurological functioning, routine MRI, and MEG recordings. Healthy controls were included in the Amsterdam MS cohort for a case-controlled comparison (Tewarie et al., 2014a).

### Technical Details and Recording Protocol of the MEG-Recordings

**Figure 1:**
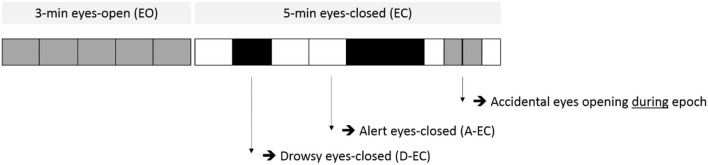
Example of the scoring of epochs (vertical lines mark the start/end of different epochs, each epoch is 13.1072 s) for both the eyes-open (EO) part (part one, light gray) and the eyes-closed (EC) part (part two, white and black) of the recording protocol. Epochs in part two were scored for the presence of alert eyes-closed (A-EC, white) or drowsy eyes-closed (D-EC, black) epochs. Note the accidental opening of the eyes during the eyes-closed condition.

Magnetoencephalography data were acquired using a 306-channel whole-head MEG system (Elekta Neuromag Oy, Helsinki, Finland), while participants were in supine position inside a magnetically shielded room (VacuumSchmelze GmbH, Hanua, Germany). The recording protocol consisted of 3-min eyes-open followed by a 5-min recording with the eyes closed in the majority of subjects. Refer to Figure 1 for a schematic overview of the recording protocol. In case of clear signs of imminent sleep in the ongoing recording (such as slow rolling eye movements or clear slowing of the posterior dominant rhythm), an auditory stimulus was given by the laboratory technician to arouse the subject. Horizontal eye movements were measured using EOG electrodes placed to the left and right of the eye.

A sample frequency of 1,250 Hz was used. An anti-aliasing filter of 410 Hz and a high-pass filter of 0.1 Hz were applied online, and other artifacts were removed offline using the temporal extension of signal space separation (tSSS) in MaxFilter software with a sliding window and correlation limit of 10 and 0.9, respectively (Elekta Neuromag Oy, version 2.2.10) (Taulu et al., 2004; Taulu and Simola, 2006; Medvedovsky et al., 2009). Before tSSS, malfunctioning channels were removed after careful visual inspection of the raw data [SK]. The mean number of excluded channels was 5.4, (range: 1–10). The participants' head position in relation to the MEG sensors was continuously recorded using signals from four head localization coils. The head localization coil positions were digitized, as well as the outline of the participants scalp (~500 points), using a 3D digitizer (Fastrak, Polhemus, Colchester, VT).

Scalp surfaces of all subjects were co-registered to their structural MRIs using a surface-matching procedure, with an estimated resulting accuracy of 4 mm (Whalen et al., 2008). The automated anatomical labeling (AAL) atlas was used to define 78 cortical regions of interest (ROIs) (Gong et al., 2009). Broadband (0.5–70 Hz) time series were estimated for the centroid of each of these ROIs using an atlas-based beamforming approach described previously (Hillebrand et al., 2012, 2016). Specifically, an equivalent current dipole was used as a source model, and a single sphere, which was fitted to the outline of the scalp as obtained from the co-registered MRI, was used as a volume conductor model. A scalar beamformer implementation (beamformer, version 2.1.28; Elekta Neuromag Oy) similar to synthetic aperture magnetometry (Robinson and Vrba, 1999) was used to compute broadband beamformer weights, which were subsequently normalized (Cheyne et al., 2007). Broadband data were used for the computation of the beamformer weights, singular value truncation (with the default setting of 1e-06 times the maximum singular value) was used when inverting the data covariance matrix to deal with the rank deficiency of the data after tSSS (~70 components), and a unity noise covariance matrix was used for the estimation of the optimum source orientation using singular value decomposition (Sekihara et al., 2004). Inspection of source-space data and further analyses of the time series were done with the in-house developed software package Brainwave (version 0.9.152.12.26), Available from http://home.kpn.nl/stam7883/brainwave.html.

### Epoch Selection: Eyes-Open, Alert Eyes-Closed, and Drowsy Epochs

Each of the 66 subjects was screened (ES) for the presence of an equal number of non-overlapping eyes-closed (A-EC), eyes-open (EO), and drowsy (D-EC) artifact-free epochs of 4,096 samples (13.1072 s, downsampled by factor 4) (Fraschini et al., 2016).

Eyes-open (EO) epochs were selected from the first 3 min of the recording. They were characterized by the absence of a clearly visible posterior dominant “background” pattern and the presence of eye-blink artifacts (mainly visible in signal space). The other epochs were selected from the second part of the recording (refer to Figure 1). Alert eyes-closed (A-EC) resting state was defined as epochs with a clear posterior dominant background pattern present (in most subjects an alpha rhythm), absence of clear (eye-blink) artifacts, and the absence of signs of alpha dropout and/or significant slowing of the background pattern. Drowsy eyes closed (D-EC) was defined as epochs with an alpha dropout for short periods of time (<50% of epoch length), increased theta power in epochs compared to other epochs in the same recording, and/or slow, roving eye movements in the eye-movement channels, but which did not fulfill the AASM criteria for NREM1 based on the duration of these alterations (<50% alpha in 30-s epochs) (Santamaria and Chiappa, 1987; Berry et al., 2017; Asadi-Pooya and Sperling, 2019).

To retain a sufficient number of subjects with enough and a comparable number of epochs in all states, we decided to start with the selection of subjects with at least 5 epochs per condition/state per subject (15 epochs in total). A total of 19 subjects (out of the 66 healthy controls in the Amsterdam MS cohort) could be included based on this selection criterion. After the analysis of the dataset with 5 epochs, we decided to reanalyze our data with 10 epochs per subject. Unfortunately, a majority of these cases had artifacts in the eyes-open part of the recording, so only the drowsy (*n* = 10) and eyes-closed alert (*n* = 10) epochs could be used. Also, only 15 cases had 10 artifact-free epochs in the awake and drowsy states. Results of this second analysis of only 15 cases but with more data per case (10 epochs) are presented in the Supplementary Tables S2, S3.

### Example of Visual Epoch Selection and Quantitative Assessment of Epochs on a Case-by-Case Basis

**Figure 2:**
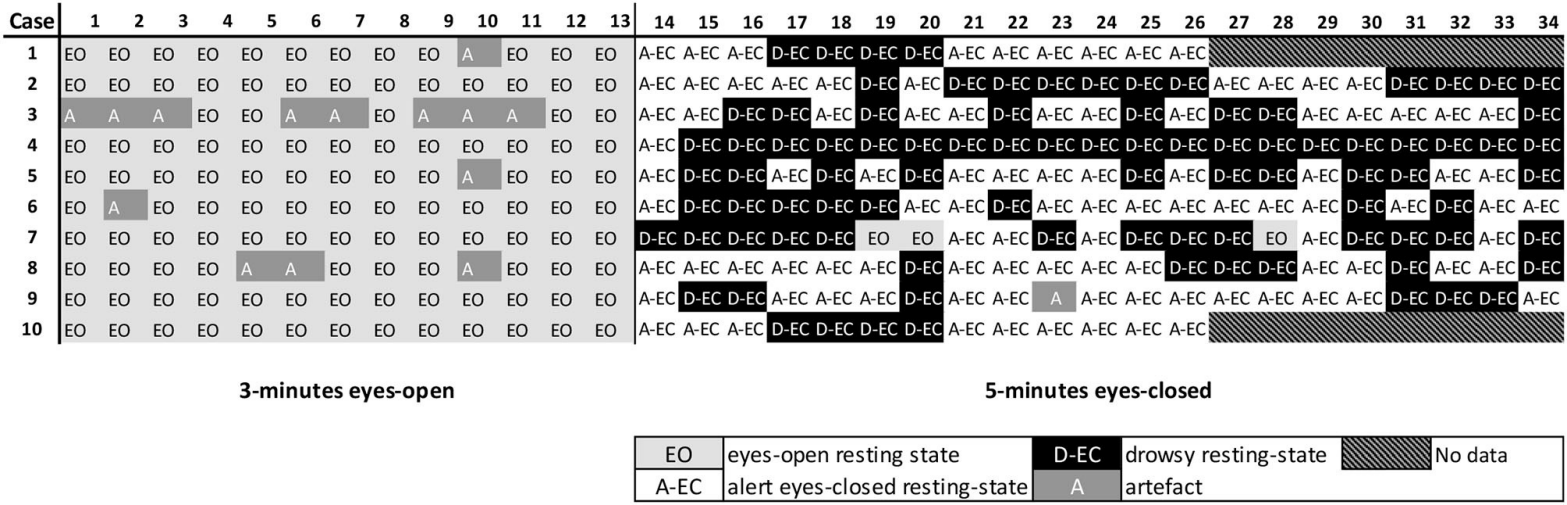
Example of the scoring of the MEG recording for the first subjects. Represented are 34 epochs (13.1072 s each) for the first 10 subjects. Subjects 1 and 10 had a shorter recording than that was defined in the protocol. Marked in white are epochs that were scored as resting-state alert eyes-closed (A-EC) epochs. Marked in black are epochs that were scored as drowsy eyes-closed (D-EC) epochs. Marked in light gray are epochs that were scored as the eyes-open (EO) epochs. Marked in a darker gray are epochs where a clear artifact was present (A). Note the presence of drowsiness in some subjects soon after the start of the eyes-closed part of the recording. Also note the opening of the eyes during the “eyes-closed” (EO) condition.

**Figure 3:**
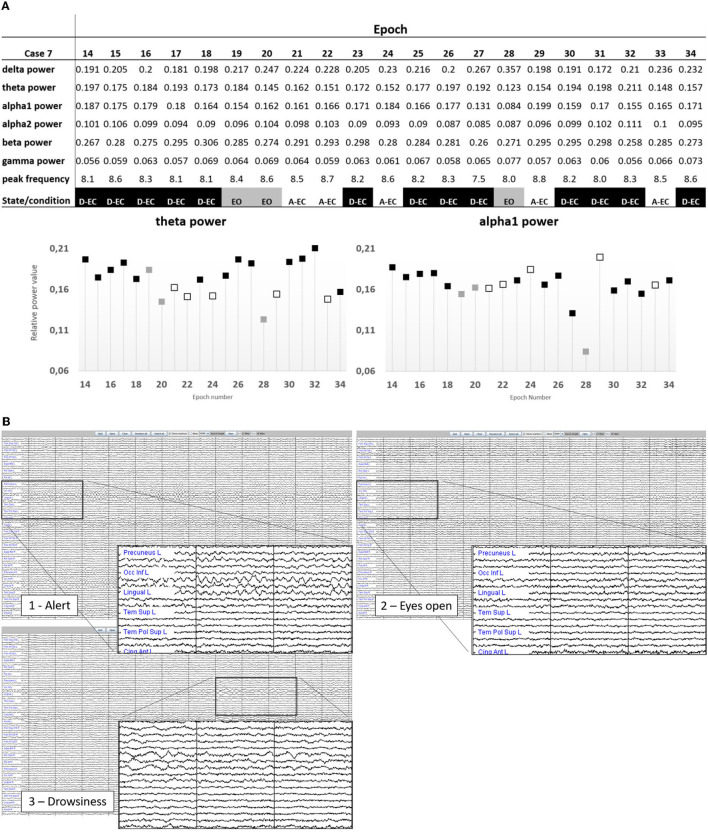
**(A)** Representation of the state changes in subject one of the subjects. Note the changes in the power values depending on the presence of drowsiness, or visual suppression of the background pattern (epoch 28 in the eyes-open condition). Only epochs 21, 22, 24, 29, and 33 were without any signs of drowsiness. Epoch length was 13.1072 s. **(B)** Source-space time-series visualized in Brainwave (epoch length 4,096 samples, sample frequency of 312 Hz, resulting in an epoch length of 13.1072 s). Shown are state changes in subject 7. Image 1 is epoch 29 of subject 7 (example of an A-EC epoch). In image 2 (epoch 28), the subject has opened the eyes (despite the instruction to keep them closed). In image 3 (epoch 30), there is clear slowing of the background pattern indicative of drowsiness/sleep. The magnifications show 3 s of the AAL regions 21 (left precuneus) to 30 (left anterior cingulate).

Refer to Figures 2, 3 for examples of the epoch scoring for a fraction of the subjects (Figure 2) and 1 subject individually (Figure 3). Figure 3 shows how time series change in appearance and shows the fluctuations of relative theta and alpha1 power over time/epochs in the different states. There is a clear suppression of the dominant background pattern with a drop in theta or alpha1 power when the subject opened the eyes (epoch 28). These effects are also present, but to a lesser extent, in epochs 18–19. Of note, here is that epochs with a duration of 13 s were used: quite often changes in state or condition (eyes opening) occurred during an epoch, so that the effects on relative power measures tend to average out. Refer to Figure 1 for a theoretical example.

### Time Series Analyses

For each subject, time series was digitally divided into the six classical EEG/MEG frequency bands [delta (0.5–4 Hz), theta (4–8 Hz), alpha1 (8–10 Hz), alpha2 (10–13 Hz), beta (13–30 Hz), and gamma (30–48 Hz)] by fast Fourier transform of the data; setting activity outside passband to zero, followed by inverse Fourier transform (brickwall filter). This resulted in 6 sets of 78 time series (i.e., 6 sets for each cortical ROI). From these time series, the relative power for each band and peak frequency (frequency with the maximum power in the 4–13 Hz range) were calculated. Global cortical relative power for the different frequency bands was constructed as mean relative power over all 78 cortical ROIs and 5 epochs (or 10 epochs for dataset 2). Similarly, average peak frequency was estimated as the mean peak frequency over all 78 cortical ROIs and 5 (or 10) epochs. Refer to Table 1 for a schematic overview of analyzed variables.

**Table 1:** Results of spectral and connectivity analyses for the eyes-closed alert (A–EC) and drowsy state (D–EC), and eyes-open (EO) condition.

**5 epochs** **(**~**13 s)**	**Eyes-closed alert** **(A-EC)**		**Eyes-open resting state (EO)**		**Eyes-closed drowsy (D-EC)**		***p*** **-value**		Regional difference?^*^	
**FFT**	**Mean**	**SD**	**Mean**	**SD**	**Mean**	**SD**	**A-EC vs. EO**	**A-EC vs. D-EC**	**A-EC vs. EO**	**A-EC vs. D-EC**
Delta power	0.269	(0.043)	0.313	(0.061)	0.283	(0.042)	<10^−7^	0.0001	**Yes**	**Yes**
Theta power	0.137	(0.015)	0.141	(0.019)	0.151	(0.017)	<0.001	<10^−7^	**Yes**	**Yes**
Alpha1 power	0.089	(0.021)	0.073	(0.017)	0.086	(0.021)	<10^−7^	0.110	Yes	No
Alpha2 power	0.125	(0.024)	0.097	(0.022)	0.112	(0.020)	<10^−7^	<10^−7^	**Yes**	**Yes**
Beta power	0.303	(0.057)	0.295	(0.061)	0.289	(0.049)	<0.01	<10^−7^	**Yes**	**Yes**
Gamma power	0.078	(0.010)	0.081	(0.013)	0.078	(0.012)	0.001	0.501	No	No
Peak frequency	8.6	(0.6)	7.8	(0.9)	8.0	(0.6)	<10^−7^	<10^−7^	**Yes**	**Yes**
**PLI**										
Delta (0.5–4 Hz)	0.113	(0.004)	0.113	(0.003)	0.112	(0.003)	0.513	0.236	No	No
Theta (4–8 Hz)	0.095	(0.004)	0.095	(0.004)	0.096	(0.005)	0.672	0.105	No	No
Alpha1 (8–10 Hz)	0.138	(0.009)	0.136	(0.008)	0.138	(0.012)	0.295	0.823	No	No
Alpha2 (10-13 Hz)	0.117	(0.010)	0.111	(0.007)	0.113	(0.007)	0.074	0.098	No	No
Beta (13–30 Hz)	0.051	(0.003)	0.051	(0.003)	0.051	(0.003)	0.507	0.064	No	No
Gamma (30-48 Hz)	0.048	(0.003)	0.048	(0.003)	0.049	(0.003)	0.727	0.711	No	No
**AECc**										
Delta (0.5–4 Hz)	0.504	(0.009)	0.520	(0.021)	0.505	(0.015)	0.005	0.896	**Yes**	No
Theta (4–8 Hz)	0.508	(0.013)	0.511	(0.015)	0.512	(0.016)	0.695	0.081	No	No
Alpha1 (8–10 Hz)	0.517	(0.019)	0.518	(0.021)	0.518	(0.023)	0.968	0.872	No	No
Alpha2 (10–13 Hz)	0.522	(0.023)	0.523	(0.024)	0.524	(0.028)	0.778	0.999	No	No
Beta (13–30 Hz)	0.518	(0.014)	0.520	(0.017)	0.520	(0.018)	0.856	0.481	No	No
Gamma (30–48 Hz)	0.501	(0.003)	0.502	(0.003)	0.502	(0.004)	0.586	0.189	No	No

*FFT, fast Fourier transform; PLI, phase lag index; AECc, corrected amplitude envelope correlation. Presented p-values are not corrected for multiple comparisons for the differences between states*.

* *Regional differences: defined as “no” if none of the 78 cortical AAL ROIs had a significant (p < 0.05 after correction for multiple comparisons using FDR) between states*.

### Functional Connectivity and Network Topology

Subsequently, we estimated functional connectivity between 78 AAL regions using two frequently used methods; the phase lag index (PLI) (Stam et al., 2007) and corrected amplitude envelope correlation (AECc) (Hipp et al., 2012). The PLI is a phase-based measure with correction for volume conduction. It estimates the asymmetry of the distribution of phase differences between time series. The AECc is based on the amplitude envelope correlation (AEC) (Bruns et al., 2000) and uses pairwise orthogonalization prior to the calculation of the AEC to correct for volume conduction. The AEC in turn is an amplitude-based measure that estimates the Pearson correlation between the envelopes of the amplitude of time series. The amplitude envelops are calculated using the Hilbert transform of the time series. See also more detail, including a study of reproducibility, of both measures (amongst others) by Briels et al. (2020).

Global FC was calculated by averaging all values in the matrix. Regional FC was analyzed as average FC from 1 ROI to all other ROIs. For each epoch and each participant separately, the minimum spanning tree (MST) subgraph was constructed using the PLI or AECc connectivity matrix (Tewarie et al., 2014b). This resulted in a dichotomized backbone of the functional brain network formed by the 78 cortical regions and 77 strongest functional connections, as the MST contains a fixed number of regions (i.e., nodes) and connections (i.e., links). Consequently, there are no arbitrary thresholds, which optimizes comparability between subjects and states or conditions (Stam et al., 2014). The two extreme tree topologies exist: (1) a line-like tree where all nodes are connected to two other nodes with the exception of the two, the so-called, leaf nodes at either end that have only one link, and (2) a star-like tree where all leaves are connected to one central node. There are many different tree types between these two extremes (Boersma et al., 2013).

The tree topology can be characterized by various measures, and global MST network measures are informative about the functional integration and segregation of the entire network (Boersma et al., 2013; Tewarie et al., 2015). Here, we used the leaf fraction (LF), the tree hierarchy (TH), and betweenness centrality (BC). Leaf fraction is a measure based on the leaf number, which is defined as the number of nodes that have only one connection. It ranges between 2 (a line topology; such a tree is called a path) and a maximum value *M* = *n* – 1 (with *n* the number of nodes) (a star-like topology). Leaf fraction is the leaf number divided by the maximum possible leaf number: *L*_f_ = *L*/*M*. Tree hierarchy (TH) characterizes a hypothesized optimal topology of the efficient organization while preventing information overload of central nodes. For a line-like topology *T*_h_ = 0, for a star-like topology *T*_h_ = 0.5, and for trees with a configuration between these 2 extreme situations, *T*_h_ can have values of *T*_h_ → 1. BC of a node *u* is defined as the number of shortest paths between any two nodes *i* and *j* in the network that are passing *u*, divided by the total number of shortest paths. BC ranges between 0 (leaf node) and 1 (central node in a star-like network). The BC of the tree was characterized by the BC_max_, i.e., the BC of the node with the highest BC in the tree. Nodes with a high BC are considered “hub nodes” not only based on their number of connections, but also on their importance for global communication in the network. Maximum BC describes the importance of the most central node, which is a measure of central network organization (van Dellen et al., 2018).

### Statistical Analysis

Analyses were done on global and regional levels. For each subject, peak frequency was calculated for each ROI in each epoch and then averaged over the available epochs to get regional values. A global value was obtained by subsequently averaging over all 78 ROIs. Similarly, power values for each of the 6 different frequency bands were calculated for each ROI and epoch, averaged over epochs to obtain regional values, and subsequently averaged over all ROIs to obtain global values. Global and regional FC were calculated as described in paragraph 2.4. The MST metrics LF, TH, and BC were averaged over epochs.

Differences between average values per subject or group (for peak frequency, power values, connectivity values, and MST measures) and individual ROIs per subject or group (for regional power and FC differences and regional differences in BC) were compared between the 3 different states or conditions (EO, A-EC, and D-EC) using the pairwise *t*-tests. Regional results were corrected for 78 comparisons (78 AAL ROIs) using the false discovery rate (FDR). An alpha <0.05 after the correction was considered statistically significant. All analyses were performed for the dataset with all states (set of 19 subjects with EO, A-EC, and D-EC data) as well as for the dataset with more epochs, but fewer cases (subjects with A-EC and D-EC data). All statistical analyses were performed in IBM SPSS Statistics 26 and Microsoft Excel 2016.

## Results

### Descriptives and Availability of Data

Time series of 66 healthy controls were screened for the presence of at least 5 epochs in the alert eyes-closed, drowsy eyes-closed, and eyes-open states or conditions. Recordings were on average of 35 epochs long and consisted of 13 epochs in the EO state for all of the subjects (by design) and a median of 22 epochs (range 13–33 epochs) for the eyes-closed state. The eyes-closed epochs (1,448 epochs in 66 subjects) were then scored for the presence of the alert (A-EC) and drowsy (D-EC) states. There were 793 (55%) drowsy epochs and 544 (37%) alert epochs. We excluded 97 (6%) epochs with a clear artifact (mainly frequent eye-blinking in the EO condition) and 14 (1%) epochs with spontaneous eyes opening during the eyes-closed condition. Figure 2 shows an example of the scoring for the first 10/66 subjects.

Ultimately, only 19 subjects had sufficient epochs (*n* = 5) in each state or condition, so a total of 15 epochs were included per case. There were 13 women and 6 men. The mean age was 40.3 (SD 8.4) years. As also mentioned in the Section Methods, we additionally analyzed how many cases of these 19 subjects we could use if we were to include 10 epochs in each state. Then, only 15 cases [11 women and 4 men, mean age 40.9 (SD 7.1) years] had sufficient epochs in the awake and drowsy states (*n* = 10). Results of this second analysis of only 15 cases but with more data per case are presented in the Supplementary Tables S2, S3.

### The Effects of State and Condition on Spectral Power

The results of spectral analysis are presented in Table 1. Clear and significant differences between the different states (A-EC and D-EC) and different conditions (EO vs. A-EC) were present. For the eyes-open vs. eyes-closed condition, most striking differences were a lower peak frequency, lower relative alpha2 and alpha1 power, and higher relative delta power during the eyes-open (EO) condition.

Similar differences were present between the alert (A-EC) and drowsy (D-EC) states. Relative theta power was significantly higher in D-EC epochs vs. A-EC epochs whereas alpha2 power, beta power, and peak frequency were significantly lower in drowsy epochs. A second analysis [fewer subjects but more epochs (10 instead of 5)] in the alert and drowsy states confirmed these findings (refer to Table 2).

**Table 2:** Network analyses (using the minimum spanning tree (MST) in all frequency bands for the eyes-closed alert (A-EC) and drowsy states (D-EC), and eyes-open (EO) condition for 19 subjects with 5 epochs (epoch length of 13.1072 s) for each of the states or conditions.

**(A)**																
	**MST Leaf fraction (LF)**								**MST Tree hierarchy (TH)**							
**5 epochs** **(~13 s)**	**Eyes-closed alert** **(A-EC)**		**Eyes-open** **(EO)**		**Eyes-closed drowsy** **(D-EC)**		***p*** **-value**		**Eyes-closed alert** **(A-EC)**		**Eyes-open** **(EO)**		**Eyes-closed drowsy** **(D-EC)**		***p*** **-value**	
**PLI**	**Mean**	**SD**	**Mean**	**SD**	**Mean**	**SD**	**A-EC vs. EO**	**A-EC vs. D-EC**	**Mean**	**SD**	**Mean**	**SD**	**Mean**	**SD**	**A-EC vs. EO**	**A-EC vs. D-EC**
Delta (0.5–4 Hz)	0.515	(0.039)	0.512	(0.040)	0.513	(0.039)	0.600	0.800	0.384	(0.043)	0.381	(0.041)	0.382	(0.041)	0.561	0.496
Theta (4–8 Hz)	0.514	(0.039)	0.521	(0.034)	0.522	(0.039)	0.161	0.295	0.379	(0.039)	0.393	(0.037)	0.390	(0.039)	0.042	0.063
Alpha1 (8–10 Hz)	0.509	(0.041)	0.517	(0.038)	0.517	(0.038)	0.221	0.199	0.380	(0.040)	0.388	(0.043)	0.388	(0.038)	0.06	0.09
Alpha2 (10–13 Hz)	0.521	(0.041)	0.510	(0.033)	0.517	(0.035)	0.063	0.534	0.383	(0.044)	0.378	(0.033)	0.384	(0.038)	0.461	0.815
Beta (13–30 Hz)	0.514	(0.037)	0.521	(0.338)	0.520	(0.039)	0.161	0.295	0.379	(0.039)	0.393	(0.038)	0.390	(0.039)	0.012	0.067
Gamma (30–48 Hz)	0.502	(0.040)	0.509	(0.042)	0.495	(0.097)	0.237	0.553	0.375	(0.043)	0.381	(0.040)	0.385	(0.043)	0.164	0.396
**AECc**																
Delta (0.5–4 Hz)	0.497	(0.041)	0.502	(0.048)	0.482	(0.041)	0.015	0.387	0.375	(0.039)	0.364	(0.0387)	0.364	(0.038)	0.223	0.227
Theta (4–8 Hz)	0.497	(0.042)	0.500	(0.039)	0.506	(0.480)	0.568	0.129	0.368	(0.039)	0.371	(0.043)	0.373	(0.042)	0.712	0.634
Alpha1 (8–10 Hz)	0.505	(0.043)	0.495	(0.038)	0.494	(0.042)	0.098	0.061	0.377	(0.041)	0.365	(0.042)	0.364	(0.041)	0.061	0.053
Alpha2 (10–13 Hz)	0.516	(0.041)	0.508	(0.045)	0.515	(0.042)	0.164	0.831	0.375	(0.038)	0.375	(0.045)	0.382	(0.056)	0.937	0.690
Beta (13–30 Hz)	0.540	(0.046)	0.544	(0.051)	0.553	(0.057)	0.501	0.118	0.391	(0.045)	0.396	(0.043)	0.405	(0.557)	0.488	0.084
Gamma (30–48 Hz)	0.480	(0.040)	0.485	(0.043)	0.484	(0.040)	0.468	0.361	0.364	(0.034)	0.366	(0.037)	0.365	(0.041)	0.736	0.366
**(B)**									
	**MST maximum betweenness centrality (BC)**																
**5 epochs** **(~13 s)**	**Eyes-closed alert (A-EC)**				**Eyes-open (EO)**				**Eyes-closed drowsy (D-EC)**				***p*** **-value**				**Regional differences BC**
**PLI**	**Mean**		**SD**		**Mean**		**SD**		**Mean**		**SD**		**A-EC vs. EO**		**A-EC vs. D-EC**		
Delta (0.5–4 Hz)	0.675		(0.060)		0.676		(0.053)		0.678		(0.060)		0.856		0.786		No
Theta (4–8 Hz)	0.680		(0.054)		0.667		(0.059)		0.669		(0.050)		0.102		0.112		No
Alpha1 (8–10 Hz)	0.673		(0.057)		0.670		(0.060)		0.669		(0.053)		0.341		0.343		No
Alpha2 (10–13 Hz)	0.684		(0.058)		0.676		(0.055)		0.677		(0.059)		0.355		0.365		No
Beta (13–30 Hz)	0.680		(0.054)		0.667		(0.059)		0.669		(0.050)		0.083		0.112		No
Gamma (30–48 Hz)	0.673		(0.061)		0.671		(0.054)		0.669		(0.057)		0.813		0.717		No
**AECc**									
Delta (0.5–4 Hz)	0.675		(0.057)		0693		(0.063)		0.665		(0.056)		0.028		0.421		No
Theta (4–8 Hz)	0.677		(0.058)		0.679		(0.064)		0.683		(0.061)		0.825		0.480		No
Alpha1 (8–10 Hz)	0.672		(0.055)		0.684		(0.067)		0.686		(0.063)		0.202		0.092		No
Alpha2 (10–13 Hz)	0.675		(0.063)		0.673		(0.058)		0.679		(0.068)		0.229		0.103		No
Beta (13–30 Hz)	0.693		(0.065)		0.690		(0.067)		0.688		(0.064)		0.720		0.406		No
Gamma (30–48 Hz)	0.662		(0.049)		0.665		(0.055)		0.666		(0.055)		0.700		0.526		No

*FFT, fast Fourier transform; PLI, phase lag index; AECc, corrected amplitude envelope correlation. Presented p-values are not corrected for multiple comparisons for the differences between states*.

^*^Regional differences: defined as “no” if none of the 78 cortical AAL ROIs had a significant (p < 0.05 after correction for multiple comparisons using FDR) between states.

**Figure 4:**
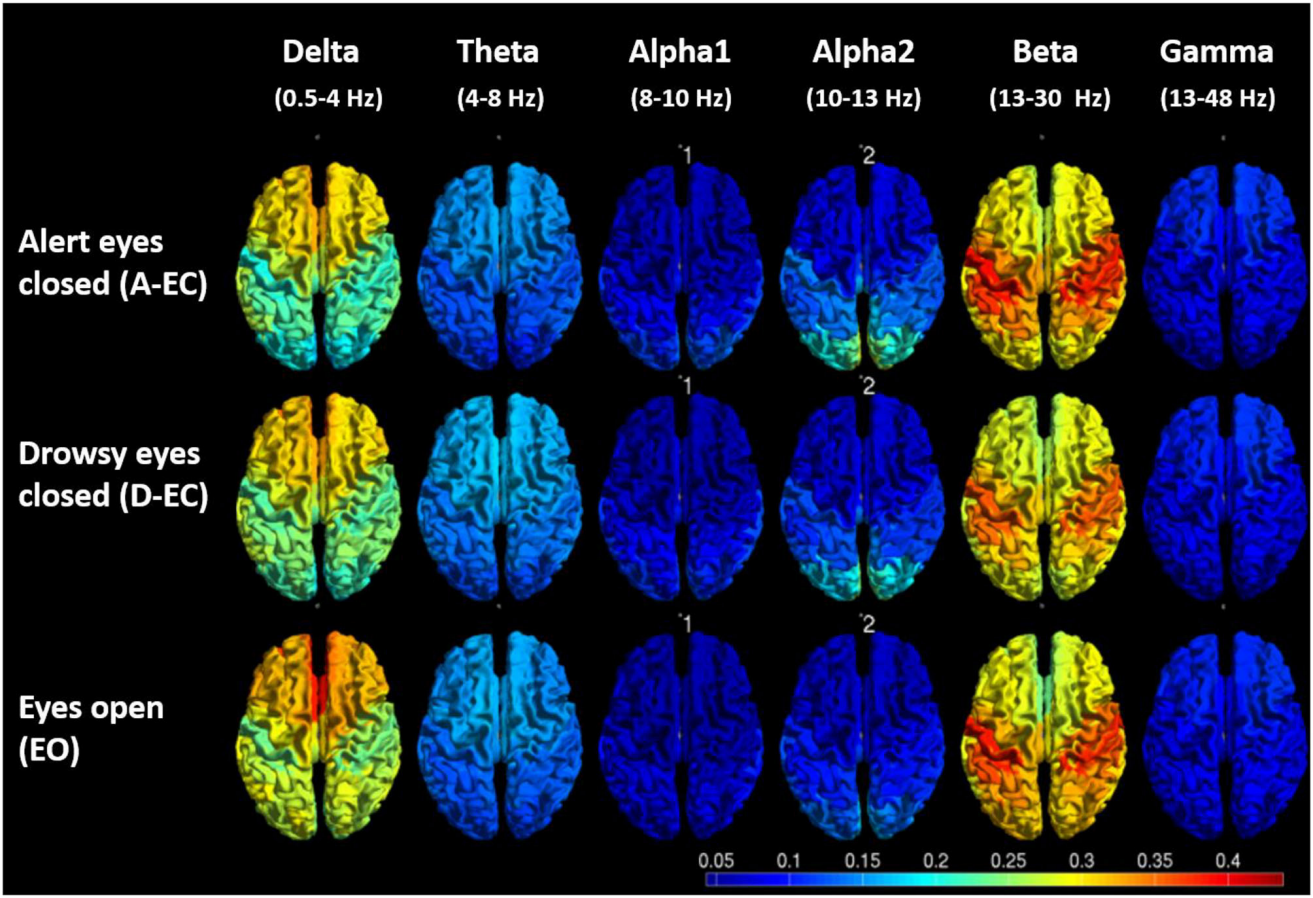
Visual representation of the average relative power values (5 epochs for 19 subjects) for different frequency bands and different conditions/states. Note the lower posterior alpha2 power EO vs. A-EC, the higher frontal delta power EO vs. A-EC, the lower central beta power D-EC vs. A-EC and higher central and frontal theta in D-EC vs. A-EC.

Not surprisingly, the differences in spectral power also translated to regional differences (i.e., the comparison of all 78 AAL ROIs between states, refer to Supplementary Table S1). There were clear differences between EO and A-EC, as well as between A-EC and D-EC, in some of the 78 cortical AAL regions in the delta, alpha2 and beta bands, and between the majority of the 78 cortical AAL regions in the theta band and in the peak frequency analysis in both datasets. Figure 4 gives a visual overview of the spatial distribution of relative power values for the different states or conditions (dataset 1).

### The Effects of State on Functional Connectivity and Network Topology; Drowsiness (D-EC) vs. Alert (A-EC) State

There were no significant differences between the A-EC and D-EC states for the connectivity analyses (PLI and AECc) and the studied MST metrics (based on the PLI and AECc) in both datasets (refer to Tables 1, 2). Repeated analysis in the sample with fewer cases but more epochs confirmed these findings (refer to Supplementary Tables S2, S3).

### The Effects of Condition on Functional Connectivity and Network Topology; Eyes Opening (EO) vs. Alert Eyes-Closed (A-EC) Condition

There were no differences in functional connectivity with the PLI between the A-EC and EO groups. Regarding network topology, however, there was a significantly higher MST TH in the theta and beta bands in the EO condition using the PLI.

There was only a difference in the AECc in the delta band between the EO and A-EC conditions. Opened eyes were related to a significantly higher delta band connectivity with the AECc. These differences also translated to changes in network topology. There was a higher LF and a higher BC in the EO condition. There were no differences in the tree hierarchy. Delta band regional BC did not differ between both states (refer to Table 2A, B; Supplementary Table S3).

## Discussion

This study investigated the effects of drowsiness and opened or closed eyes on neurophysiological measures of activation, connectivity, and network topology. Drowsiness was present in a large part of eyes-closed resting-state epochs. There were significant spectral changes between alert (A-EC) and drowsy (D-EC) states and between the eyes-open (EO) and eyes-closed conditions. Estimates of functional connectivity only differed for the AECc in the delta band between the eyes-open (EO) and eyes-closed (A-EC) conditions. Additionally, there were changes in network topology between eyes-open (EO) and eyes-closed (A-EC) conditions. Drowsiness was of far less influence on connectivity metrics or network properties.

State fluctuations in subjects during resting-state recordings are a commonly encountered phenomenon (Lal and Craig, 2002; Marquetand et al., 2019; Li et al., 2020). It is even present during repetitive activity, such as driving (Dkhil et al., 2015; Sriraam et al., 2016). In fact, in our case, it led to having far less subjects available for analysis than initially anticipated since it was present in 55% of epochs in 66 healthy controls. Drowsiness is a challenge for a clinical interpretation of EEG/MEG results since such physiological changes can easily resemble pathology. Although EEG/MEG is an excellent tool for identifying physiological changes between wakefulness and sleep, the dynamic transition between the two states and the concomitant EEG/MEG changes are difficult to define. Our study observed the effects of drowsiness on spectral power in the majority of frequency bands and in a large number of epochs. In the literature, the most recognizable characteristics of drowsiness or sleep have been described as a decreasing alpha power, an alpha dropout, an increase in theta power, and slow, horizontal eye movements in EOG channels (Santamaria and Chiappa, 1987). The AASM criteria define the first sleep phase (NREM1) as >50% of a 30-s epoch containing low-amplitude mixed-frequency EEG and/or the appearance of any of the following phenomena: (a) EEG activity in a range of 4–7 Hz with slowing of background frequencies by ≥1 Hz from those of the awake stage, (b) vertex sharp waves, and (c) slow, roving eye movements (Berry et al., 2017). However, periods of drowsiness can also be short (<50% of a 30-s epoch) and these short periods can be frequently present. Clinically, these short episodes can even be accompanied by decreased responsiveness. This often comes unnoticed by the participant. Poudel et al. (2014) showed in a task-based fMRI/EEG study that “microsleeps” (i.e., brief (0.5–15 s) episodes of complete failure to respond, accompanied by slow eye closures and EEG theta activity) were present in a surprisingly large majority of healthy participants while lying in an MRI scanner. These subjects had no unusual sleep pattern for the week prior to the scan. A similar study by Tagliazucchi showed that 30% of subjects do not maintain wakefulness (according to the AASM criteria) in the first 3 min in a resting state in an MRI scanner (de Pasquale et al., 2010; Poudel et al., 2014; Tagliazucchi and Laufs, 2014). This study confirmed that a large number of epochs (55%) can be classified as drowsy without fulfilling the criteria for NREM1 sleep.

Now, the most important question is whether the changes in condition or state matter in terms of the influence on spectral analysis or estimates of functional connectivity and network topology. In the extensive body of literature on intra-individual variability in spectral power, the main focus is on test–retest reliability, but drowsiness is often not specifically taken into account (Craig et al., 2012; Babiloni et al., 2021). Previous studies reported that the power density of resting-state EEG recordings in healthy controls is stable at 12–40 months retest (Napflin et al., 2007; Duan et al., 2021). There is a high test–retest correlation (*r* = 0.84 at 12–14 weeks; intra-class correlation coefficients = 0.8–0.9 at 4 weeks), and the absolute and relative power densities are quite consistent when computed from artifact-free time series of 20 s to 4 min (Salinsky et al., 1991). Relative power densities seem slightly more repeatable (Duan et al., 2021). Also, reproducibility of spectra EEG measurements over recording sessions seems better in eyes-closed (EC) settings than in eyes-open settings possibly due to the absence of eye-blink artifacts. This perhaps suggests that on average, drowsiness may not be a very significant problem. However, when specifically selecting data on drowsiness, there are important and significant differences especially in the power spectrum as we have shown here. These changes we observed are in line with the previous work that most often shows a shift in oscillatory activity to the lower frequency bands (i.e., theta and alpha 1 bands) (Yeo et al., 2009).

It is interesting that we found that connectivity measures (AECc and PLI) and MST metrics are far less—if at all—influenced by state changes. Previous work in EEG suggested differences in coherence between sleep stages (Kaminski et al., 1997). Our study indicated that effects on functional connectivity were absent for PLI, and AECc effects were only seen in the delta band for eyes open (EO) vs. eyes closed (A-EC). Data of patients with Alzheimer's disease show that the reproducibility of EEG/MEG results is dependent on the type of connectivity measure and frequency band; the AECc is most robust in the alpha and beta frequency bands and the PLI in the theta band (Colclough et al., 2016; Briels et al., 2020). It needs to be mentioned that the AECc and PLI may measure different types of connectivity and are in a sense complementary to each other. There is no satisfactory explanation for the meaning and importance of the different results both measures sometimes give. We, therefore, chose to study both measures.

Marquetand et al. additionally showed that vigilance does not influence overall test–retest reliability. However, they did not study the connectivity metrics that we used in our work *per se* and only compared selected awake epochs instead of the effects of drowsiness specifically. Possibly, both the AECc and the PLI are too robust “trait” measures [i.e., stable in individuals (Demuru et al., 2017), regardless of state] or too noisy to detect subtle changes. The differences we found in the AECc in the delta band and subsequently in some of MST metrics (i.e., in the network topology) may be caused by less subtle effects, such as eye movements (i.e., eye blinks), as suggested by Bodala et al. (2015).

As in all studies, there are some limitations to our work. First of all, the classification of drowsiness remains arbitrary since it involves a dynamic state between wakefulness and sleep and no clear criteria for its presence exist. Nevertheless, the differences we found between the drowsy and awake states in the spectral analysis were highly significant and clinically relevant. So even with such a highly dynamic state, attention to its presence makes a clear difference. There is also the methodological issue of the common source effect (Palva et al., 2018). Both the PLI and AECc are however harbor corrections for field spread. So, it is highly unlikely that common source effects are of importance here. Additionally, we agree that the number of subjects and number of epochs are fairly limited. We screened an extensive number of healthy controls (*n* = 66) for the presence of sufficient drowsy, awake, and eyes-open epochs. We felt it to be important that sufficient data were available for all three states to be able to properly assess inter-individual differences with the appropriate pairwise tests. Ultimately, only 19/66 subjects had enough epochs in all 3 states. We do however believe that it is possible to draw valid conclusions on this subset. The states clearly differ in terms of the spectral analysis (virtually all frequency bands differ highly significantly in terms of relative power). In addition, although there is a significantly altered relative power, connectivity measures remain rather stable or unchanged. Additionally, a very recent (2022) publication by Wiesman et al. shows that the stability of spectral resting-state MEG measurements can be robustly estimated in most cortical regions when derived from relatively short segments of 30–120 s of resting-state data. We have used a minimum of 5^*^13 s (Wiesman et al., 2021).

As far as the translation to other datasets is concerned, this study used manual epoch selection, which is labor-intensive, especially for larger sample sizes. Recently, algorithmic approaches to monitor alertness and drowsiness, such as a blind source separation method—independent component analysis (ICA)—have been applied to EEG data (Hsu and Jung, 2017). ICA is already a widely used approach to eliminate assorted artifacts. However, the application of ICA for the distinction between alert and drowsy transitions is relatively new. One issue is that individual ICA components may, or may not, be attributable to drowsiness. ICA may also influence subsequent connectivity estimates, resulting in added variation in results. This approach may however be of value if used for the automatic detection of epochs that should be excluded from subsequent analyses.

Ultimately, the awareness of drowsiness being present, even within seconds after the start of a recording, is important especially when the goal is to perform spectral power analyses. Also, conditions can change unexpectedly (i.e., the subject can open his/her eyes even when instructed otherwise), and this has the potential to influence brain connectivity studies significantly. It is of note that we found many of these events in healthy controls, and it may be even more extensively present in persons with neurological diseases that lead to cognitive disturbances. Epoch selection by trained raters is therefore of utmost importance, especially when performing spectral analyses. For functional connectivity analyses using the AECc and/or PLI, as well as studies on network topology, it seems mainly important to assess whether subjects really held to the eyes-closed/eyes-open instructions. Also, for future studies into neurodegenerative diseases, especially when connectivity metrics are used other than the AECc/PLI, it is important to test the influence of state to rule out any physiological effect coming from the spectral changes that are the result of drowsiness.

## Conclusion

Drowsiness during eyes-closed resting-state MEG/EEG recordings was present in the majority of epochs. This had considerable influence on spectral power but not on connectivity and network topology. These findings are important for future studies that use EEG/MEG to study the dynamics of neurological disease. Recordings should be evaluated for the presence of drowsiness when spectral analyses are performed. For connectivity analyses or studies on changes in network topology, this is far less important.

## Data Availability Statement

The raw data supporting the conclusions of this article will be available to qualified researchers upon request from the corresponding author. The MEG time-series cannot be shared publicly due to privacy regulations regarding data sharing outside of the European Union.

## Ethics Statement

The studies involving human participants were reviewed and approved by METc Amsterdam UMC, Locatie VUmc. The patients/participants provided their written informed consent to participate in this study.

## Author Contributions

ES, YT, AH, and CS conceptualized the study, IN, SK, DS, LR, and MS collaborated, performed the data analysis, interpreted the results, wrote the first draft, reviewed, and edited the manuscript for intellectual content. DS, LR, and MS collaborated in interpreting the results and in reviewing and editing the manuscript for intellectual content. All authors contributed to the article and approved the submitted version.

## Conflict of Interest

MS has received compensation for consulting services or speaker honoraria from ExceMed, MedDay, Atara, Sanofi-Genzyme, and Biogen. The remaining authors declare that the research was conducted in the absence of any commercial or financial relationships that could be construed as a potential conflict of interest.

## Publisher's Note

All claims expressed in this article are solely those of the authors and do not necessarily represent those of their affiliated organizations, or those of the publisher, the editors and the reviewers. Any product that may be evaluated in this article, or claim that may be made by its manufacturer, is not guaranteed or endorsed by the publisher.
