# Supplementary material for: State Changes During Resting-State (Magneto)encephalographic Studies: The Effect of Drowsiness on Spectral, Connectivity, and Network Analyses
Source: Front Neurosci. 2022 Jun 14;16:782474. doi: 10.3389/fnins.2022.782474 (PMC9245543; doi:10.3389/fnins.2022.782474)
Supplement: Supplementary file 1 [file Data_Sheet_1.docx]

**Supplementary table S1 |** Regions with significant differences in the power spectrum between the eyes closed (A-EC) and drowsy (D-EC) state.

|  | **AAL regions with significant spectral power differences between the A-EC and D-EC group** | |
| --- | --- | --- |
| **Frequency band** | **Left** | **Right** |
| **Delta** | Occipital inf L |  |
|  |  | Cuneus R |
|  | Fusiform L |  |
|  | Heschl L | Heschl R |
| **Theta** | **all 78 AAL regions except for the middle orbitofrontal gyrus left and right** | |
| **Alpha1** | Heschl L |  |
| **Alpha2** | Rectus L |  |
|  | Olfactory L |  |
|  | Frontal Sup Orb L | Frontal Sup Orb R |
|  | Frontal Med Orb L |  |
|  | Frontal Sup Medial L | Frontal Sup Medial R |
|  |  | Precuneus R |
|  |  | Occipital Sup R |
|  | Occipital Mid L | Occipital Mid R |
|  | Occipital Inf L | Occipital Inf R |
|  |  |  |
|  | Calcarine L |  |
|  | Cuneus L | Cuneus R |
|  | Lingual L | Lingual R |
|  | Fusiform L | Fusiform R |
|  |  | Heschl R |
|  |  | Temporal Sup R |
|  |  | Temporal Mid R |
|  |  | Temporal Inf R |
|  |  | Temporal Pole Sup R |
|  |  | ParaHippocampal R |
|  |  | Cingulum Ant R |
|  | Cingulum Post L | Cingulum Post R |
|  | Insula L | Insula R |
| **Beta** | Rectus L | Rectus R |
|  | Postcentral L |  |
|  |  | SupraMarginal R |
|  |  | Angular R |
|  | Precuneus L |  |
|  | Temporal Sup L |  |
|  | Temporal Mid L |  |
|  | Temporal Inf L |  |
|  | Temporal Pole Sup L |  |
|  | Temporal Pole Mid L |  |
|  | Cingulum Post L |  |
| **Gamma power** | No regional differences | |
| **Peak frequency** | Rectus L |  |
|  | Frontal Sup Orb L |  |
|  | Frontal Med Orb L |  |
|  | Frontal Inf Orb L |  |
|  | SupraMarginal L | SupraMarginal R |
|  |  | Occipital Sup R |
|  |  | Fusiform R |
|  |  | Heschl R |
|  | Temporal Sup L |  |
|  | Temporal Mid L |  |
|  | Temporal Pole Sup L | Temporal Pole Sup R |
|  |  | Cingulum Ant R |
|  | Insula L | Insula R |

**Table S1 |** Regions with significant differences between the different vigilance states (A-EC and D-EC). ROIs with p<0.05 (after FDR correction for 78 regions) were considered statistically significant. Most striking are the differences in theta power (all regions) and alpha2 power. Of note is that the regions that are not symmetrically altered in the alpha2 band (mainly the temporal regions) are also found to be asymmetrically different in the beta band.

**Supplementary S2 |** Results of spectral and connectivity analyses in all frequency bands for the eyes-closed alert (A-EC) and drowsy state (D-EC) analyzed using a larger number of epochs per state (n=10 epochs for n=15 subjects)

| **10 epochs**  **(~13 seconds)** | **Eyes-closed alert (A-EC)** | | **Eyes-closed drowsy**  **(D-EC)** | | **p-value** | **Significant* regional difference?**  **(any of the 78 AAL regions)** |
| --- | --- | --- | --- | --- | --- | --- |
| **FFT** | **Mean** | **(SD)** | **Mean** | **SD** | **A-EC vs D-EC** | **A-EC vs D-EC** |
| delta power | 0.270 | (0.045) | 0.278 | (0.052) | **0.013** | **Yes** |
| theta power | 0.138 | (0.012) | 0.158 | (0.033) | **<10^-7^** | **Yes** |
| alpha1 power | 0.096 | (0.026) | 0.094 | (0.031) | 0.228 | **Yes** |
| alpha2 power | 0.118 | (0.022) | 0.107 | (0.018) | **<10^-7^** | **Yes** |
| beta power | 0.301 | (0.047) | 0.286 | (0.049) | **<10^-7^** | **Yes** |
| gamma power | 0.077 | (0.011) | 0.077 | (0.016) | 0.245 | No |
| peak frequency | 8.5 | (0.5) | 7.9 | (0.6) | **<10^-7^** | **Yes** |
| **PLI** |  | | | | | |
| Delta (0.5-4 Hz) | 0.112 | (0.008) | 0.115 | (0.007) | 0.063 | No |
| Theta (4-8 Hz) | 0.095 | (0.005) | 0.096 | (0.006) | 0.462 | No |
| Alpha1 (8-10 Hz) | 0.137 | (0.010) | 0.139 | (0.011 | 0.389 | No |
| Alpha2 (10-13 Hz) | 0.115 | (0.006) | 0.113 | (0.006) | 0.102 | No |
| Beta (13-30 Hz) | 0.048 | (0.003) | 0.049 | (0.003) | 0.918 | No |
| Gamma (30-48 Hz) | 0.048 | (0.004) | 0.049 | (0.005) | 0.943 | No |
| **AECc** |  | | | | | |
| Delta (0.5-4 Hz) | 0.506 | (0.012) | 0.508 | (0.020) | 0.061 | No |
| Theta (4-8 Hz) | 0.505 | (0.010) | 0.507 | (0.014) | 0.101 | No |
| Alpha1 (8-10 Hz) | 0.522 | (0.029) | 0.523 | (0.025) | 0.295 | No |
| Alpha2 (10-13 Hz) | 0.528 | (0.030) | 0.527 | (0.032) | 0.270 | No |
| Beta (13-30 Hz) | 0.519 | (0.019) | 0.520 | (0.017) | 0.166 | No |
| Gamma (30-48 Hz) | 0.502 | (0.004) | 0.503 | (0.006) | 0.406 | No |

**Table S2 |** Results of frequency and connectivity analyses in all frequency bands for the alert and drowsy state, in 15 subjects with 10 epochs (epoch length of 13.1072 seconds) for each of the states/conditions. Alpha1 = 8-10 Hz, alpha2 10-13 Hz. FFT = Fast Fourier Transform, PLI = phase lag index, AECc corrected amplitude envelope correlation, NS = not significant. Regional differences: defined as no if none of the 78 cortical AAL ROIs had a significant (p<.05 after correction for multiple comparisons using FDR) difference in connectivity or betweenness centrality (BC) value between states.

**Supplementary S3 |** Results of the network analyses in all frequency bands for the eyes-closed alert (A-EC) and drowsy state (D-EC) analyzed using a larger number of epochs per state (n=10 epochs for n=15 subjects)

|  | **MST leaf fraction (LF)** | | | | | **MST tree hierarchy (TH)** | | | | | **MST betweenness centrality (BC)** | | | | | |
| --- | --- | --- | --- | --- | --- | --- | --- | --- | --- | --- | --- | --- | --- | --- | --- | --- |
| **10 epochs**  **(~13 seconds)** | **Eyes-closed alert (A-EC)** | | **Eyes-closed drowsy (D-EC)** | | **p-value** | **Eyes-closed alert (A-EC)** | | **Eyes-closed drowsy (D-EC)** | | **p-value** | **Eyes-closed alert (A-EC)** | | **Eyes-closed drowsy (D-EC)** | | **p-value** | **Regional differences BC?** |
| **PLI** | **Mean** | **SD** | **Mean** | **SD** | **A-EC vs D-EC** | **Mean** | **SD** | **Mean** | **SD** | **A-EC vs D-EC** | **Mean** | **SD** | **Mean** | **SD** | **A-EC vs D-EC** |  |
| Delta (0.5-4 Hz) | 0,513 | (0.039) | 0.517 | (0.038) | 0.426 | 0.383 | (0.041) | 0.388 | (0.046) | 0.408 | 0.673 | (0.057) | 0.672 | (0.056) | 0.916 | No |
| Theta (4-8 Hz) | 0.512 | (0.036) | 0.514 | (0.039) | 0.632 | 0.379 | (0.038) | 0.384 | (0.042) | 0.024 | 0.671 | (0.057) | 0.683 | (0.056) | 0.061 | No |
| Alpha1 (8-10 Hz) | 0.518 | (0.040) | 0.525 | (0.041) | 0.213 | 0.387 | (0.041) | 0.392 | (0.040) | 0.433 | 0.673 | (0.057) | 0.673 | (0.053) | 0.966 | No |
| Alpha2 (10-13 Hz) | 0.526 | (0.046) | 0.521 | (0.042) | 0.285 | 0.387 | (0.045) | 0.390 | (0.045) | 0.599 | 0.684 | (0.064) | 0.672 | (0.061) | 0.093 | No |
| Beta (13-30 Hz) | 0.512 | (0.04) | 0.513 | (0.037) | 0.828 | 0.384 | (0.040) | 0.386 | (0.038) | 0.662 | 0.670 | (0.053) | 0.666 | (0.047) | 0.520 | No |
| Gamma (30-48 Hz) | 0.506 | (0.037) | 0.501 | (0.037) | 0.259 | 0.380 | (0.044) | 0.377 | (0.041) | 0.585 | 0.670 | (0.058) | 0.668 | (0.058) | 0.879 | No |
| **AECc** |  | | | | | | | | | | | | | | | |
| Delta (0.5-4 Hz) | 0.489 | (0.035) | 0.490 | (0.043) | 0.785 | 0.365 | (0.039) | 0.367 | (0.041) | 0.595 | 0.675 | (0.059) | 0.671 | (0.053) | 0.543 | No |
| Theta (4-8 Hz) | 0.488 | (0.036) | 0.488 | (0.042) | 0.998 | 0.367 | (0.040) | 0.379 | (0.043) | 0.859 | 0.682 | (0.059) | 0.679 | (0.060) | 0.769 | No |
| Alpha1 (8-10 Hz) | 0.498 | (0.072) | 0.503 | (0.045) | 0.468 | 0.372 | (0.039) | 0.366 | (0.058) | 0.279 | 0.675 | (0.097) | 0.679 | (0.057) | 0.666 | No |
| Alpha2 (10-13 Hz) | 0.519 | (0.076) | 0.519 | (0.042) | 0.916 | 0.381 | (0.041) | 0.378 | (0.062) | 0.595 | 0.684 | (0.058) | 0.681 | (0.101) | 0.718 | No |
| Beta (13-30 Hz) | 0.552 | (0.056) | 0.540 | (0.082) | 0.117 | 0.390 | (0.064) | 0.401 | (0.049) | 0.106 | 0.693 | (0.061) | 0.687 | (0.104) | 0.568 | No |
| Gamma (30-48 Hz) | 0.489 | (0.042) | 0.485 | (0.045) | 0.295 | 0.365 | (0.043) | 0.369 | (0.041) | 0.449 | 0.664 | (0.057) | 0.668 | (0.056) | 0.887 | No |

**Table S3 |** Results of network analyses in all frequency bands for the alert and drowsy state, in 15 subjects with 10 epochs for each of the states/conditions. FFT = Fast Fourier Transform, PLI = phase lag index, AECc corrected amplitude envelope correlation. Regional differences: defined as “no” if none of the 78 cortical AAL ROIs had a significant (p<.05 after correction for multiple comparisons using FDR) difference in connectivity or betweenness centrality (BC) value between states.
